# Oxidative balance score and depression in COPD patients: A cross-sectional study from NHANES 2007 to 2018

**DOI:** 10.1097/MD.0000000000046004

**Published:** 2025-12-12

**Authors:** Ke Chen, Bowen Xu, Lu Zhang, Li Fang, Di Wu, Mengyao Shi, Zegeng Li

**Affiliations:** a The First Clinical Medical College of Anhui University of Chinese Medicine, Hefei, China; b Institute of Traditional Chinese Medicine for Respiratory Disease Prevention and Treatment, Anhui Academy of Traditional Chinese Medicine, Hefei, China; c Anhui Provincial Key Laboratory of Translational Medicine for Prevention and Treatment of Major Respiratory Diseases with Traditional Chinese Medicine, Hefei, China; d Anhui University of Chinese Medicine, Hefei, China; e Anhui Provincial Chinese Medicine Hospital, Hefei, China.

**Keywords:** chronic obstructive pulmonary disease, depression, diet, lifestyle, oxidative balance score

## Abstract

Chronic obstructive pulmonary disease (COPD) is a prevalent chronic respiratory condition often associated with inflammation and metabolic dysregulation. Depressive symptoms show a notably higher prevalence among individuals with COPD compared with the general population (Hegerl U, Mergl R. Depression and suicidality in COPD: understandable reaction or independent disorders? Eur Respir J. 2014;44:734–43). Oxidative stress has been identified as a critical shared pathological mechanism underlying both COPD and depression. However, the association between the oxidative balance score (OBS) and depression in COPD patients remains underexplored. This study analyzed data from the National Health and Nutrition Examination Survey (NHANES) spanning 2007 to 2018. The sample included 1020 COPD patients, 169 of whom were diagnosed with depression (PHQ-9 score ≥ 10). OBS, calculated based on 20 dietary and lifestyle factors, was assessed for its association with depression risk using weighted logistic regression and restricted cubic spline models. Analyses were stratified by gender to examine subgroup differences. Depression prevalence among COPD patients was 16.56%. A significant negative association was observed between OBS and depression risk, with higher OBS quartiles correlating with lower depression risks (adjusted OR for the highest quartile: 0.290, 95% CI: 0.193–0.434, *P* < .001). Stratified analysis demonstrated that female patients and non-Hispanic White individuals experienced greater benefits. Dietary OBS was found to have a stronger protective effect on depression risk compared to lifestyle OBS. Higher OBS scores were significantly associated with reduced depression risk in COPD patients, particularly among women and specific demographic subgroups. These findings underscore the importance of antioxidant-rich diets and healthy lifestyle choices in promoting mental health among COPD patients. The results provide a foundation for personalized intervention strategies and call for further research to validate the causal mechanisms underlying OBS.

## 1. Introduction

Chronic obstructive pulmonary disease (COPD) is a common chronic respiratory condition characterized by airflow limitation, often accompanied by exacerbated inflammatory responses and various metabolic disorders.^[1,2]^ In recent years, the psychological health issues of COPD patients have garnered increasing attention, particularly the high prevalence of depression, which poses significant challenges to patients’ quality of life and disease management^[3]^ statistics indicate that approximately 20 to 50% of COPD patients experience varying degrees of depression, a proportion markedly higher than that in the general population.^[3,4]^ The bidirectional relationship between COPD and depression is complex and multifaceted, potentially involving chronic inflammation, oxidative stress, metabolic imbalances, and psychosocial factors.^[5,6]^

Oxidative stress, as a critical shared pathological mechanism of COPD and depression, has attracted considerable academic attention in recent years.^[7]^ Under conditions of oxidative stress, the balance between pro-oxidant and antioxidant factors is disrupted, resulting in sustained oxidative damage and inflammatory responses in cells and tissues.^[8]^ This imbalance not only exacerbates the pathological progression of COPD but may also trigger or worsen depression through inflammatory mediators and neural pathways^[9]^ Oxidative stress is closely related to various lifestyle and dietary factors, such as smoking, alcohol consumption, unhealthy eating habits, and insufficient physical activity.^[10]^

The oxidative balance score (OBS) is a composite measure of oxidative stress levels, calculated by weighting scores of antioxidants and pro-oxidants from dietary and lifestyle factors.^[11,12]^ Higher OBS values indicate a dominant antioxidant state, which may confer protective effects against oxidative stress-related diseases. Previous studies have demonstrated significant negative correlations between OBS and diseases such as cardiovascular disease, chronic kidney disease, and type 2 diabetes.^[13–15]^ However, the role of OBS in COPD patients, particularly its relationship with psychological health, remains insufficiently studied.

Against this background, this study utilized data from the National Health and Nutrition Examination Survey (NHANES) 2007 to 2018 to explore the association between OBS and depression in COPD patients through cross-sectional analysis. The study aimed to elucidate the potential protective effects of OBS on the mental health of COPD patients and to further clarify the role of oxidative stress in the dual burden of COPD and depression. By analyzing NHANES 2007 to 2018 data, the study investigated the relationship between OBS and depression in COPD patients and examined the potential modifying effects of chronic disease status on this relationship. Stratified analyses were conducted to reveal the differential effects of OBS in patients with and without chronic diseases, providing scientific evidence for psychological health management in COPD patients and offering a reference for developing personalized dietary and lifestyle interventions.

## 2. Materials and methods

### 2.1. Data source and study population

This study analyzed data from the 2007 to 2018 cycles of the National Health and Nutrition Examination Survey (NHANES), investigating the relationship between the OBS and depression in patients with COPD. COPD was defined as either a self-reported physician diagnosis of COPD or emphysema, or a post-bronchodilator forced expiratory volume in one second/ forced vital capacity ratio below 0.7.^[4,16]^ The NHANES data collection protocol was approved by the National Center for Health Statistics Ethics Review Board. Written informed consent was obtained from all participants, and data were anonymized, so no additional ethical review was required. NHANES employed a multi-stage stratified probability sampling design to ensure nationally representative data.

**Figure 1.**
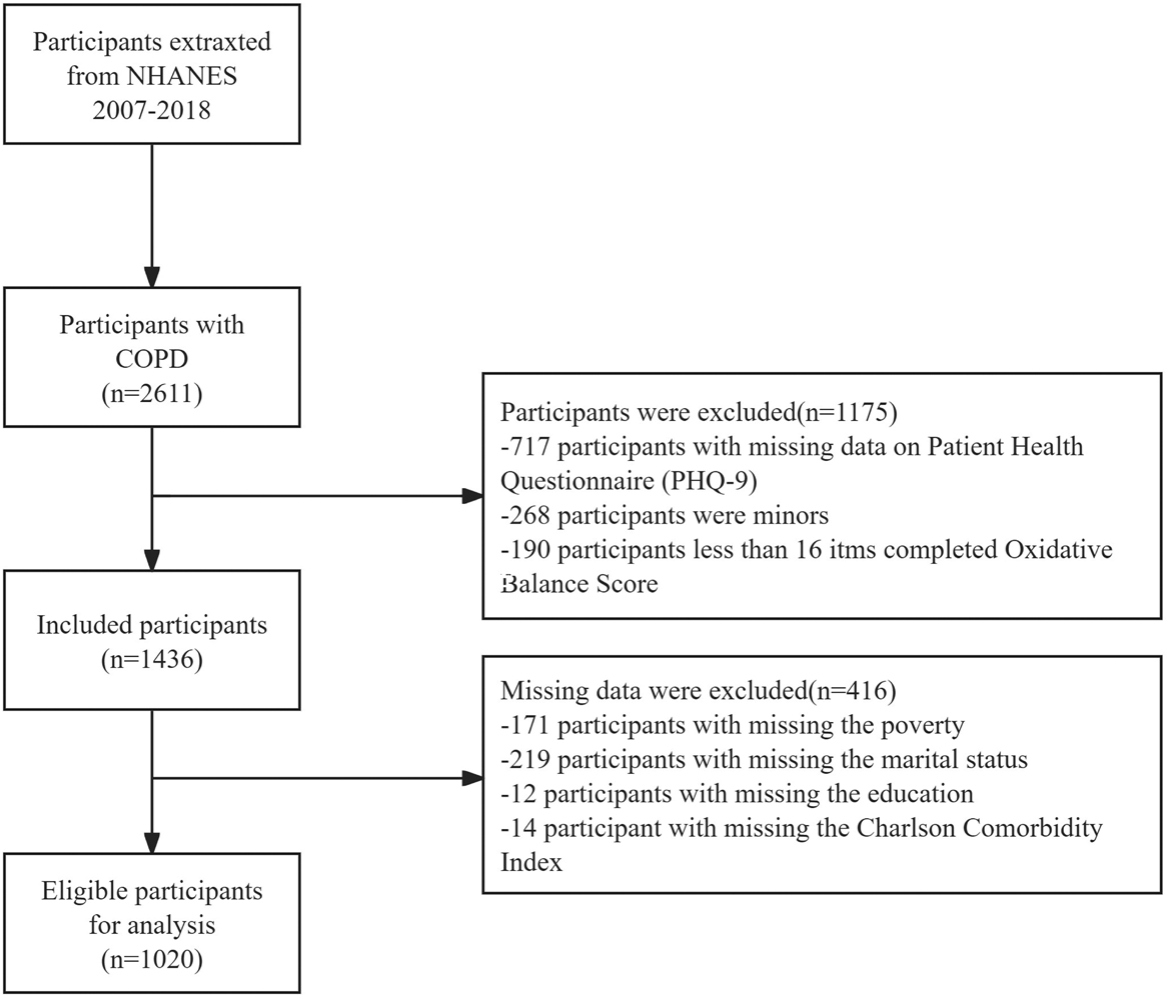
Flowchart of the sample selection from NHANES 2007–2018. NHANES = National Health and Nutrition Examination Survey.

A total of 2611 COPD participants were initially identified. Of these, 1175 were excluded for the following reasons: 717 lacked data from the patient health questionnaire-9 (PHQ-9), 268 were under 20 years old, and 190 had incomplete OBS data (fewer than 16 completed items). An additional 416 participants were excluded due to missing key variables, including 171 lacking poverty-to-income ratio (PIR) data, 219 missing marital status data, 12 missing education level data, and 14 missing Charlson comorbidity index (CCI) data. Ultimately, 1020 participants met the inclusion criteria and were analyzed, as illustrated in Figure 1.

### 2.2. Calculation of the oxidative balance score (OBS)

OBS was calculated using 24-hour dietary recall interviews and included 16 dietary components: dietary fiber, carotenoids, riboflavin, niacin, vitamin B6, total folate, vitamin B12, vitamin C, vitamin E, calcium, magnesium, zinc, copper, selenium, total fat, and iron.^[14,17]^ Two interviews were conducted: the first in person and the second via telephone 3 to 10 days later. Only the data from the first interview were used in this study to ensure the reliability of self-reported dietary intake and minimize confounding due to changes in physiological or health status over time.

Lifestyle OBS was based on factors including physical activity, body mass index (BMI), alcohol consumption, and smoking status. Total fat, iron, BMI, alcohol, and smoking were categorized as pro-oxidants, while other variables were considered antioxidants. Physical activity scores were calculated according to NHANES guidelines using metabolic equivalent (MET) values, incorporating work-related activities (vigorous and moderate), leisure-time activities (vigorous and moderate), and walking or cycling for transportation. MET scores were computed as: weekly frequency × duration per session × MET value for each activity. Smoking status was assessed using serum cotinine levels (a primary metabolite of nicotine).

Alcohol consumption was classified into 3 groups based on Zhang et al^[11]^ heavy drinking (≥15 g/d for women, ≥30 g/d for men), non-heavy drinking (0–15 g/d for women, 0–30 g/d for men), and non-drinking, with scores of 0, 1, and 2 assigned respectively. OBS was calculated by assigning scores based on sex-specific tertiles. Antioxidants were scored from 0 (lowest tertile) to 2 (highest tertile), while pro-oxidants were scored in reverse (highest tertile = 0). Total OBS scores ranged from 0 to 40. This study used total OBS, dietary OBS, and lifestyle OBS as variables, with detailed compositions provided in Table S1 (Supplemental Digital Content, https://links.lww.com/MD/Q689).

### 2.3. Depression assessment

Depression was assessed using the PHQ-9, a validated self-reported diagnostic tool based on Diagnostic And Statistical Manual criteria, which includes 9 depressive symptoms.^[18]^ Participants rated the frequency of each symptom over the past 2 weeks on a 4-point scale: 0 (not at all), 1 (several days), 2 (more than half the days), and 3 (nearly every day). The total scores ranged from 0 to 27. A PHQ-9 total score ≥ 10 was used to define depression in this study. This threshold is widely adopted in clinical and epidemiological studies to indicate depressive symptoms,^[19–22]^ with a sensitivity and specificity of 88% as validated in clinical settings.^[18]^

### 2.4. Covariate assessment

Covariates were selected based on prior research and relevant hypotheses, encompassing sociodemographic, nutritional, and lifestyle factors, as well as comorbidities. Sociodemographic variables included age, sex, race/ethnicity (categorized as Mexican American, non-Hispanic White, non-Hispanic Black, other Hispanic, and other races), educational attainment (less than high school, high school or above), marital status (classified as single, married/living with a partner, or other), and the PIR, grouped as <1.3, 1.3–3.5, and ≥3.5.

Nutritional factors were assessed using total energy intake, the Healthy Eating Index (HEI_2015), and dietary OBS. Lifestyle factors included smoking status, alcohol consumption, BMI, and physical activity levels, quantified using metabolic equivalent tasks. Health status was evaluated using the CCI, which measures the number and severity of comorbid conditions. Depression status was assessed using the PHQ-9.

By systematically categorizing these variables, this study ensured a comprehensive adjustment for potential confounders in the analysis of OBS and its association with depressive symptoms in COPD patients.

### 2.5. Data analysis

To investigate the relationship between the OBS and depression in patients with COPD, this study utilized generalized linear models and weighted logistic regression.^[23]^ OBS served as the independent variable, while depression status was the dependent variable. Adjustments were made for confounders, including age, sex, BMI, race, PIR, marital status, and education level. The analysis accounted for the complex sampling design of NHANES by incorporating sampling weights, stratification, and clustering. Weighted odds ratios (ORs) and 95% confidence intervals (CIs) were reported, with statistical significance set at *P* < .05.

To assess heterogeneity in the OBS-depression relationship, stratified analyses were conducted based on key demographic and health-related factors such as BMI, sex, race, marital status, PIR, and comorbidity status. Logistic regression models were used to calculate ORs and 95% CIs for each stratified group, and interaction *P*-values (*P* for interaction) were computed. To explore potential nonlinear associations, restricted cubic spline (RCS) analysis was employed, introducing OBS RCS scores into weighted logistic regression models with 4 knots. Wald tests determined the significance of overall and nonlinear trends.

The complex sampling design of NHANES was incorporated into all analyses using sampling weights (WTMEC2YR), primary sampling units, and stratification variables (SDMVSTRA) to ensure accuracy and representativeness. Sensitivity analyses were performed to validate the robustness of results, excluding extreme values (e.g., BMI < 15 kg/m^2^, BMI > 60 kg/m^2^, and OBS scores outside the 1st and 99th percentiles) and analyzing OBS trends with alternative groupings such as tertiles and quintiles. Trend coefficients and *P*-values were computed to confirm the stability of the OBS-depression relationship.

Results were presented as weighted ORs with 95% CIs, while the nonlinear association between OBS and depression was visualized using curve plots. Detailed sensitivity and stratified analyses were provided in tables and figures. All data analyses were conducted using R software (version 4.2.0). The survey package was used for complex sampling corrections, the rms package for RCS analysis, ggplot2 for data visualization, and dplyr and tidyr for data processing.

## 3. Results

### 3.1. Baseline characteristics

Table 1 summarizes the baseline characteristics of the study population, stratified by OBS quartiles. The weighted sample size was 7,639,636 individuals (unweighted: 1020). Significant differences in baseline characteristics were observed across OBS quartiles (*P* < .05).

**Table 1:** Weighted Patient demographics and baseline characteristicsof participants according to the oxidative balance score’s quartile.

Characteristic	Weighted N = 7,639,636 Unweighted n = 1020	OBS_group				*P*-value
		Q1 Weighted N = 1,610,345 Unweighted n = 268*	Q2 Weighted N = 1,985,656 Unweighted n = 254*	Q3 Weighted N = 1,961,060 Unweighted n = 239*	Q4 Weighted N = 2,082,576 Unweighted n = 259*	
Age	57 (47, 65)	57 (48, 64)	56 (48, 64)	58 (45, 64)	58 (47, 66)	.7792
Sex						
Female	46.2%	52.7%	47.3%	41.7%	44.2%	.2533
Male	53.8%	47.3%	52.7%	58.3%	55.8%	
BMI	27 (23, 32)	29 (23, 33)	27 (24, 34)	27 (23, 30)	26 (23, 30)	.0222
Race						
Mexican American	1.7%	0.6%	2.1%	1.1%	2.6%	.0303
Non-Hispanic Black	7.2%	12.1%	7.7%	4.4%	5.5%	
Non-Hispanic White	84.6%	79.8%	84.1%	87.8%	85.8%	
Other Hispanic	2.3%	2.1%	1.0%	3.6%	2.6%	
Other Race – including multi-racial	4.2%	5.5%	5.1%	3.1%	3.6%	
Marital status						
Divorced/separated/widowed	25.7%	30.9%	28.6%	21.6%	22.8%	.2823
Married/living with a partner	64.2%	57.8%	64.2%	66.2%	67.5%	
Never married	10.1%	11.3%	7.2%	12.2%	9.8%	
Educational attainment						
˂High school	18.6%	24.5%	22.4%	12.8%	15.7%	.0113
College	55.8%	45.4%	50.7%	61.7%	63.3%	
High school	25.6%	30.1%	26.8%	25.4%	21.0%	
Poverty, n (%)						
<1.3	24.5%	41.0%	25.3%	18.3%	17.0%	<.0013
≥3.5	44.3%	23.8%	45.9%	53.1%	50.4%	
1.3–3.5	31.1%	35.2%	28.7%	28.5%	32.6%	
Total energy intake kcal		1298 (1017, 1763)	1867 (1559, 2300)	2237 (1815, 2931)	2760 (2072, 3732)	<.0012
CCI, n (%)						
˂2	75.7%	74.0%	74.4%	72.0%	81.9%	.1663
≥2	24.3%	26.0%	25.6%	28.0%	18.1%	
HEI_2015	20.00 (20.00, 21.50)	20.00 (20.00, 20.00)	20.00 (20.00, 20.00)	20.00 (20.00, 22.39)	20.00 (20.00, 24.50)	.0022
Lifestyle OBS	5.00 (4.00, 6.00)	4.00 (3.00, 5.00)	5.00 (4.00, 6.00)	5.00 (4.00, 6.00)	5.00 (5.00, 6.00)	<.0012
Dietary OBS	13 (8, 19)	4 (2, 6)	10 (8, 12)	16 (14, 17)	21 (20, 23)	<.0012
Depression						
No	86.6%	75.8%	83.7%	92.2%	92.5%	<.0013
Yes	13.4%	24.2%	16.3%	7.8%	7.5%	

BMI = body mass index, CCI = Charlson comorbidity index, HEI = healthy eating index, OBS = oxidative balance score, Q = quartiles; Q1 (4,12], Q2 (12,17], Q3 (17,23], Q4 (23,35].

* Median (IQR); %.

† Kruskal–Wallis rank-sum test for complex survey samples.

‡ χ^2^ test with Rao & Scott second-order correction.

Individuals in the highest OBS quartile (Q4) displayed healthier characteristics, including a lower median BMI (Q1: 29; Q4: 26, *P* = .0222) and a higher proportion of college-educated participants (Q4: 63.3%, *P* = .0113). Regarding racial composition, African Americans were overrepresented in Q1 (12.1%), while non-Hispanic Whites predominated in Q3 and Q4 (*P* = .0303).

In terms of health-related characteristics, total energy intake significantly increased with higher OBS quartiles (Q1: 1298 kcal; Q4: 2760 kcal; *P* < .0012). The HEI scores were also higher in Q4 compared to other quartiles (*P* = .0022). Depression prevalence was highest in Q1 (24.2%) and lowest in Q4 (7.5%, *P* < .0013), suggesting that higher OBS was associated with a lower risk of depression. Supplementary analyses (Table S2, Supplemental Digital Content, https://links.lww.com/MD/Q689) indicated that females accounted for a larger proportion of the depressed group (57.3%, *P* = .018), while college-educated participants were underrepresented in the depressed group (36.8% vs 58.8%, *P* < .001). Unmarried, divorced, and impoverished individuals also had higher proportions in the depressed group. Overall, higher OBS was associated with favorable distributions in demographic and health characteristics, supporting its potential protective effect against depression.

### 3.2. Association between OBS and depression in COPD

Table 2 illustrates the association between OBS and depression risk, showing that higher OBS quartiles were significantly associated with reduced depression risk.

**Table 2:** Weighted logistic regression analysis models showing the associations between OBS and depression in patients with chronic obstructive pulmonary disease.

OBS	Crude model		Model 1		Model 2		Model 3	*P*-value
	OR 95% CI	*P*-value	OR 95% CI	*P*-value	OR 95% CI	*P*-value	OR 95% CI	
Q1	Ref		Ref		Ref		Ref	
Q2	0.61 (0.37, 1.01)	.055	0.79 (0.46, 1.36)	.369	0.68 (0.37, 1.23)	.194	0.67 (0.37, 1.23)	.191
Q3	0.27 (0.13,0.56)	<.001	0.39 (0.17, 0.87)	.022	0.28 (0.11, 0.69)	.007	0.28 (0.11, 0.69)	.006
Q4	0.25 (0.14, 0.44)	<.001	0.36 (0.21, 0.62)	<.001	0.21 (0.10, 0.43)	<.001	0.21 (0.10, 0.43)	<.001
*P* for trend		<.001		<.001		<.001		<.001

Note: Crude model: Adjusted with no covariates; Model 1: Adjusted for age, sex, race, marital status, education level, and poverty-income ratio; Model 2: Additionally adjusted for HEI, and total energy intake. The OBS converted to a categorical variable (quartiles) is consistent with Table 1.

CI = confidence interval, OBS = oxidative balance score, OR = odds ratio, Ref = reference group.

The *P* for trend represents the significance of the overall trend between increasing OBS levels and depression risk reduction.

Crude Model: In the unadjusted model, depression risk decreased significantly across increasing OBS quartiles (*P* for trend < .001). Compared to Q1, quartiles Q3 and Q4 showed significantly lower depression risks (OR = 0.27, 95% CI: 0.13–0.56, *P* < .001; OR = 0.25, 95% CI: 0.14–0.44, *P* < .001). Stepwise Adjustment Models: After adjusting for basic demographic factors (Model 1), Q3 and Q4 remained significantly associated with reduced depression risks (OR = 0.39, 95% CI: 0.17–0.87, *P* = .022; OR = 0.36, 95% CI: 0.21–0.62, *P* < .001). Further adjustments for HEI and total energy intake (Model 2), as well as comorbidities and additional covariates (Model 3), yielded consistent results. Depression risks in Q3 and Q4 were further reduced (OR = 0.28, 95% CI: 0.11–0.69, *P* = .006; OR = 0.21, 95% CI: 0.10–0.43, *P* < .001). Trend Analysis: In all models, trend analysis revealed significant results (*P* for trend < .001), confirming that higher OBS was robustly associated with lower depression risk, particularly in Q3 and Q4.

These findings demonstrate a strong negative correlation between OBS and depression risk, supporting its potential protective role in the mental health of COPD patients.

### 3.3. Association of dietary and lifestyle OBS with depression in COPD

We further explored the association between OBS and the depressive status of COPD patients by dividing OBS into dietary and lifestyle OBS, as shown in Table 3. Dietary OBS: Higher dietary OBS quartiles (Q3 and Q4) were significantly associated with reduced depression risk in both unadjusted and fully adjusted models (Q3: OR = 0.27, 95% CI: 0.13–0.56; Q4: OR = 0.25, 95% CI: 0.14–0.44, *P* < .001). After full adjustment, this protective effect persisted (Q3: OR = 0.28, 95% CI: 0.11–0.69, *P* = .006; Q4: OR = 0.21, 95% CI: 0.10–0.43, *P* < .001). Trend analysis further supported a significant negative association between dietary OBS and depression risk (*P* for trend = 0.003). Lifestyle OBS: In the unadjusted model, higher lifestyle OBS quartiles (Q2–Q4) were associated with lower depression risk (*P* < .05). However, after full adjustment, the association weakened and was no longer statistically significant (Q4: OR = 0.99, 95% CI: 0.97–1.01, *P* = .083). Trend analysis similarly showed no significant results (*P* for trend = .743). Integrated analysis: Dietary OBS consistently demonstrated significant protective effects across all models, while lifestyle OBS showed weaker associations, potentially due to residual confounding. These results indicate that dietary OBS plays a more prominent role in reducing depression risk, underscoring the need for further investigation into its specific mechanisms.

**Table 3:** Weighted logistic regression analysis models showing the associations between dietary OBS/lifestyle OBS and depression in patients with chronic obstructive pulmonary disease.

OBS	Q1	Q2 (OR 95% CI)	*P*	Q3 (OR 95% CI)	*P*	Q4 (OR 95% CI)	*P*	*P* for trend
Dietary OBS								
Crude model	Ref	0.82 (0.54, 1.24)	.342	0.39 (0.23, 0.67)	<.001	0.49 (0.31, 0.79)	.003	<.001
Model 1	Ref	0.87 (0.56, 1.34)	.525	0.45 (0.26, 0.78)	.005	0.53 (0.32, 0.87)	.012	<.001
Model 2	Ref	0.8 (0.51, 1.27)	.351	0.38 (0.21, 0.70)	.002	0.39 (0.20, 0.76)	.006	<.001
Model 3	Ref	0.84 (0.53, 1.33)	.447	0.39 (0.21, 0.72)	.002	0.4 (0.21, 0.78)	.007	<.001
Life OBS								
Crude model	Ref	0.49 (0.33, 0.71)	<.001	0.47 (0.29, 0.76)	.002	0.2 (0.08, 0.46)	<.001	<.001
Model 1	Ref	0.52 (0.35, 0.78)	.001	0.53 (0.32, 0.88)	.014	0.24 (0.1, 0.59)	.002	<.001
Model 2	Ref	0.54 (0.36, 0.81)	.003	0.54 (0.32, 0.90)	.017	0.25 (0.1, 0.6)	.002	<.001
Model 3	Ref	0.53 (0.36, 0.8)	<.001	0.55 (0.33, 0.91)	.021	0.26 (0.1, 0.63)	.003	<.001

Model 1: Adjusted for age, sex, race, marital status, education level, and poverty-income ratio; Model 2: Additionally adjusted for HEI, and total energy intake; Model 3: Additionally, adjusted for CCI.

The dietary OBS converted to a categorical variable (quartiles): Q1, [2,7]; Q2, (7,15]; Q3, (15,19]; Q4, (19,28].

The lifestyle OBS converted to a categorical variable (quartiles): Q1, [0,2]; Q2, (2,3]; Q3, (3,5]; Q4, (5,8].

CCI = Charlson comorbidity index, HEI = healthy eating index, OBS = oxidative balance score, OR = odds ratio, Ref = reference group.

### 3.4. Subgroup analysis

**Figure 2.**
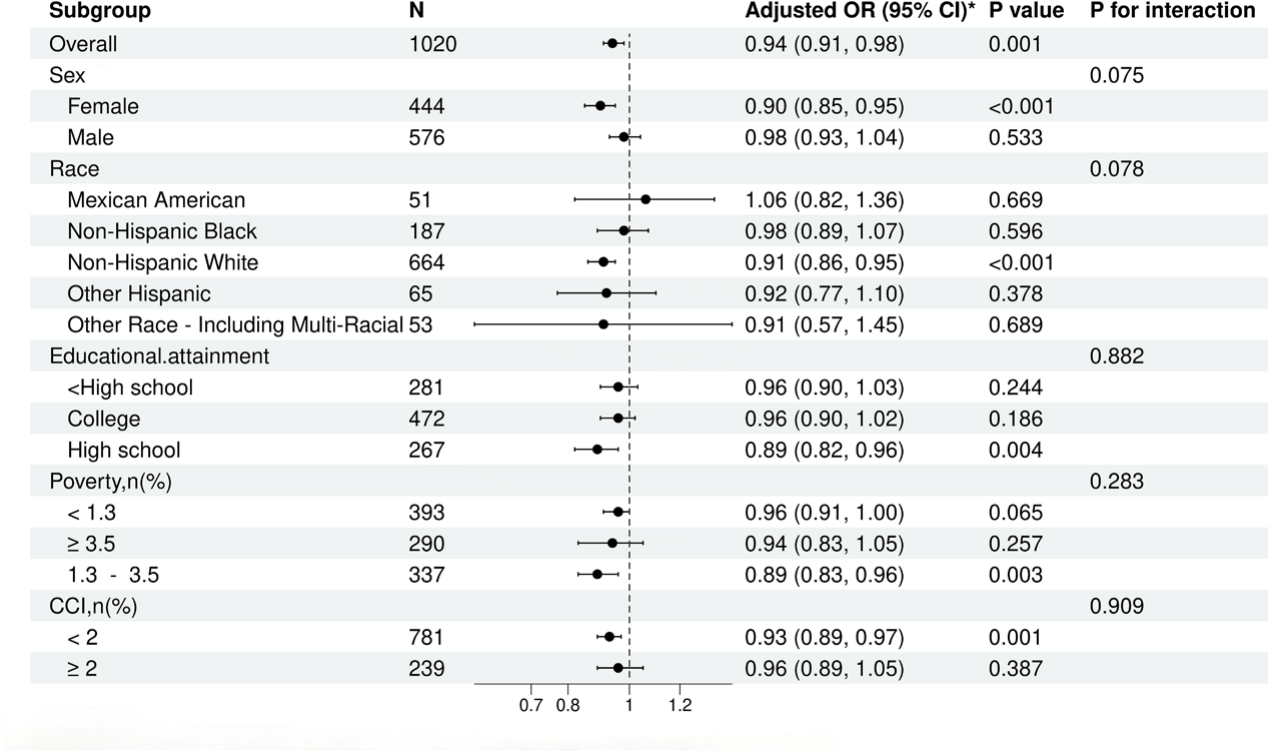
The relationship between total OBS and depression risk in chronic obstructive pulmonary disease (COPD) patients. OBS = oxidative balance score.

We used fully adjusted multivariable logistic regression models to assess the association between the OBS and depression risk in COPD patients (Fig. 2). The findings revealed that OBS significantly reduced depression risk across multiple subgroups. Among all patients, higher OBS was associated with a lower risk of depression (OR = 0.94, 95% CI: 0.91–0.98, *P* = .001).

In gender-stratified analyses, the protective effect of OBS was more pronounced in females (OR = 0.90, *P* < .001) but was not significant in males (OR = 0.98, *P* = .533). Non-Hispanic White individuals demonstrated stronger protective effects (OR = 0.91, *P* < .001), whereas other racial groups showed no significant associations.OBS also significantly reduced depression risk in patients with fewer comorbidities (CCI < 2; OR = 0.93, *P* = .001) and those with moderate poverty levels (1.3 ≤ PIR < 3.5; OR = 0.89, *P* = .003). However, no significant protective effect of OBS was observed among patients aged 65 years or older, males, or individuals categorized as “other” racial groups. While interactions between gender and education level were not statistically significant, the protective effect of OBS was more evident in females and patients with lower education levels. In summary, OBS was significantly associated with reduced depression risk in COPD patients, with the strongest effects observed in females, non-Hispanic Whites, individuals with moderate poverty levels, and those with fewer comorbidities. Future studies should investigate subgroup differences to optimize OBS-based intervention strategies.

### 3.5. Sensitivity analysis

To ensure the robustness of the results, extreme values that could potentially bias the findings were excluded. These included BMI values <15 kg/m^2^ or >60 kg/m^2^ and OBS scores outside the 1st and 99th percentiles. Regression analyses after excluding these extreme values (Table S3, Supplemental Digital Content, https://links.lww.com/MD/Q689) confirmed that the protective effect of OBS on depression risk remained significant in both unadjusted models (Crude Model) and adjusted models accounting for demographic variables (Model 1), dietary variables (Model 2), and the CCI (Model 3) (all *P*-values < .001). These findings demonstrate that the protective effect of OBS was unaffected by extreme values.

To further evaluate the impact of different grouping methods on the OBS-depression relationship (Table S4, Supplemental Digital Content, https://links.lww.com/MD/Q689), OBS was redefined into tertiles and quintiles, followed by trend analyses. The results consistently showed a significant negative association between increasing OBS and depression risk across all grouping methods. Trend coefficients were −0.50505, −0.40821, and −0.52034 for quartiles, tertiles, and quintiles, respectively (all *P* < .001). These findings confirm that the choice of grouping method did not influence the results and reinforce the protective role of OBS against depression.

Finally, the forest plot in Figure S1 (Supplemental Digital Content, https://links.lww.com/MD/Q689), visually illustrated the estimated coefficients and 95% confidence intervals of OBS across all models. None of the confidence intervals included zero, further supporting the negative association between OBS and depression risk. The results remained stable and statistically significant even after excluding extreme values.

### 3.6. Restricted cubic spline analysis

**Figure 3.**
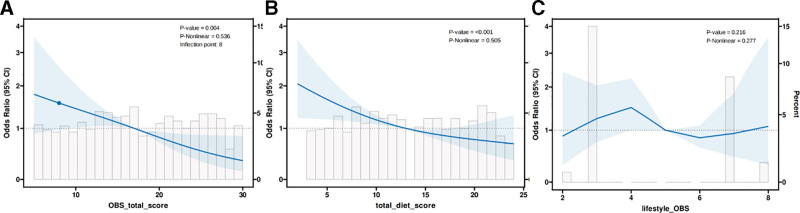
Restricted cubic spline (RCS) analysis illustrating the relationship between oxidative balance score (OBS) components and depression risk in COPD patients. (A) Total OBS shows a significant linear trend, with increasing scores associated with reduced depression risk; (B) dietary OBS demonstrates the strongest inverse association, emphasizing the role of dietary antioxidants; (C) lifestyle OBS reveals a weaker but still notable relationship, reflecting behavioral variability and residual confounding. COPD = chronic obstructive pulmonary disease.

Based on the multivariate regression analysis, RCS regression was employed to examine the relationship between total OBS, dietary OBS, lifestyle OBS, and depression risk. The analysis revealed a significant linear relationship between total OBS and depression risk, with no evidence of nonlinearity (*P* for nonlinearity = .215, Fig. 3A). Depression risk decreased significantly with increasing total OBS, highlighting the cumulative protective effects of OBS.

Dietary OBS showed the strongest inverse association with depression risk (Fig. 3B), emphasizing its role in reducing oxidative stress and systemic inflammation. In contrast, lifestyle OBS exhibited a weaker association (Fig. 3C), potentially influenced by behavioral complexity and residual confounding factors.

Overall, these findings indicate the dose-dependent protective effects of OBS, with dietary OBS being the primary contributor to reducing depression risk in COPD patients.

## 4. Discussion

This study leverages nationally representative data from the NHANES 2007 to 2018 cycle to systematically investigate the relationship between the OBS and depressive symptoms among patients with COPD. The results underscore the pivotal role of oxidative stress in the interplay between COPD and depression, revealing that a higher OBS is significantly associated with a reduced risk of depressive symptoms. This association persisted across various subgroups, including females, non-Hispanic Whites, and individuals with fewer comorbidities, providing a solid foundation for dietary and lifestyle interventions aimed at improving mental health.

### 4.1. Strong correlation between OBS and depressive symptoms in COPD

The findings highlight a robust negative correlation between OBS – a composite measure of oxidative stress – and the risk of depression in COPD patients. Specifically, individuals in the highest OBS quartile (Q4) exhibited a markedly reduced depression risk compared to those in the lowest quartile (Q1) (adjusted OR = 0.21, 95% CI: 0.10–0.43, *P* < .001; Table 2). These associations remained statistically significant even after controlling for demographic, socioeconomic, dietary, and comorbidity factors, showcasing the independent and widespread applicability of OBS in mitigating depressive symptoms.

Oxidative stress is a well-established pathological mechanism common to both COPD and depression. By exacerbating chronic lung inflammation and disrupting neuroinflammatory pathways, oxidative stress contributes to mood dysregulation through damage to neural tissues. Enhancing OBS optimizes the pro-oxidant–antioxidant balance, alleviating both systemic and neural oxidative stress. This dual mitigation likely underpins the observed reduction in depression risk (Table 2, Fig. 2).^[24]^

### 4.2. Mechanistic insights into the role of antioxidants

Antioxidants are critical in addressing oxidative stress – a shared pathological mechanism linking COPD and depression. Dietary antioxidants such as vitamins C and E, magnesium, and carotenoids play multifaceted roles in suppressing oxidative stress:

Inflammation reduction: Antioxidants mitigate systemic inflammation by suppressing pro-inflammatory cytokines, such as tumor necrosis factor-α and Interleukin-6, alleviating chronic lung inflammation and protecting brain tissues; Neurotransmitter regulation: By stabilizing serotonin and dopamine levels, antioxidants contribute to mood stabilization and improved mental health; Gut-Brain axis support: Enhancing gut microbiota diversity and reducing intestinal permeability further supports mental health through a healthy gut-brain axis. Together, these mechanisms emphasize the potential of dietary interventions to enhance OBS and reduce depression risk in COPD patients.^[25–30]^

### 4.3. Gender-specific and racial disparities in OBS’s protective effects

Stratified analyses revealed significant variability in OBS effects based on gender, race, and comorbidity status: Gender differences: OBS had a stronger protective effect in females (adjusted OR = 0.90, *P* < .001), possibly due to heightened sensitivity to oxidative stress and enhanced brain-derived neurotrophic factor signaling pathways that promote neuroplasticity and emotional regulation^[31]^; Racial disparities: Non-Hispanic Whites benefited more significantly from increased OBS, reflecting potential differences in dietary habits, lifestyle factors, and socioeconomic conditions influencing depression risk; Comorbidity-specific variability: The protective effects of OBS were more pronounced in patients with fewer comorbidities, suggesting that lower baseline oxidative stress enhances the impact of antioxidants, while severe comorbidities may attenuate these effects due to more complex pathological mechanisms.

When disaggregating OBS into dietary and lifestyle components, dietary OBS emerged as having a stronger protective effect. For instance, in fully adjusted models, the highest quartile of dietary OBS (Q4) was significantly associated with reduced depression risk (adjusted OR = 0.40, 95% CI: 0.21–0.78, *P* = .007), whereas the association for lifestyle OBS was weaker (adjusted OR = 0.26, 95% CI: 0.10–0.63, *P* = .003, Table 3). This disparity may be attributed to the direct mechanisms by which dietary antioxidants counteract oxidative stress: Direct antioxidant action of dietary components: Nutrients such as vitamin C, vitamin E, dietary fiber, and minerals (e.g., zinc, magnesium) directly neutralize reactive oxygen species, reducing oxidative stress at the molecular level. These components also possess anti-inflammatory and immune-regulating properties, enhancing gut microbiota composition and reducing systemic inflammation.^[32]^ Indirect regulation by lifestyle OBS: While smoking, alcohol consumption, BMI, and physical activity significantly impact oxidative stress, their interactions with psychosocial stressors and behavioral habits may diminish their protective effects.^[33]^ This interaction could explain the limited significance of lifestyle OBS, particularly among older adults and males, who may demonstrate greater oxidative stress tolerance and more complex pathological profiles^[34,35]^

### 4.4. Implications for subgroup-specific OBS effects

Stratified analyses revealed significant variability in the protective effects of OBS across gender, race, and health conditions. Females and non-Hispanic Whites experienced stronger benefits, likely due to differences in oxidative stress sensitivity and depression pathology. Females may exhibit greater vulnerability to oxidative stress, with higher OBS potentially providing more effective depression risk reduction through enhanced antioxidant exposure.^[36]^ Racial disparities in OBS effects may reflect variations in dietary habits, lifestyle factors, and socioeconomic conditions, which collectively influence OBS’s impact on depressive symptoms.

OBS exhibited stronger protective effects in patients with fewer comorbidities, suggesting that lower baseline oxidative stress levels may enhance its benefits. Conversely, patients with greater comorbidity burdens or severe diseases may experience attenuated OBS effects due to complex pathological mechanisms involving chronic inflammation, metabolic dysfunction, and immune suppression. These findings emphasize the importance of early intervention to maximize the protective potential of OBS.

### 4.5. Linear trends in OBS and depression risk

The RCS analysis confirmed a linear relationship between increasing OBS and reduced depression risk, with no evidence of nonlinear effects (Fig. 3). RCS analysis demonstrated a significant linear decline in depression risk with increasing total OBS, with no evidence of nonlinear effects. These findings suggest that the protective effect of OBS is cumulative rather than threshold-based.^[37,38]^ Notably, dietary OBS showed a consistent inverse association with depression risk, underscoring the substantial mental health benefits of dietary antioxidants. In contrast, lifestyle OBS did not exhibit comparable trends, reflecting its complexity and more limited role in mitigating oxidative stress.

### 4.6. Comparative context

This study builds on the findings of Zhang et al (2021), who emphasized diet as the primary determinant of OBS, demonstrating that dietary OBS exerts stronger protective effects than lifestyle OBS.^[11]^ Compared to earlier studies like Higgins et al (2020), which focused on individual antioxidant nutrients, the composite OBS framework used here integrates multiple factors, offering a more comprehensive understanding of the relationship between oxidative stress and depression.^[39]^

In conclusion, enhancing dietary OBS through targeted nutritional interventions could serve as an effective strategy to improve mental health in COPD patients, while broader applications in chronic disease management should be explored in future studies.

## 5. Strengths, limitations, and future directions

This study enhances our understanding of oxidative stress, COPD, and depression through the use of composite OBS indicators.

Strengths: comprehensive scope: integrating dietary and lifestyle factors provides a holistic view of oxidative stress’s impact on mental health, surpassing single-factor approaches; Dietary OBS validation: Supporting findings from Higgins et al (2020), this study highlights the importance of diet, particularly vitamins C and E, in reducing oxidative stress.

Limitations: lifestyle OBS variability: consistent with Zhang et al (2021), the weaker association of lifestyle OBS reflects challenges in capturing individual behavioral effects, such as smoking cessation or exercise; Observational design: The cross-sectional nature limits causal interpretations, necessitating further investigation.

Future directions: causal studies: longitudinal research and biomarker-based experiments are needed to clarify mechanisms linking OBS and depression. Behavior-specific analysis: Separate evaluation of lifestyle components like smoking and physical activity could refine understanding of their contributions; Broader impact: Exploring OBS effects in comorbid conditions may inform integrated chronic disease management strategies.

This study underscores the potential of enhancing dietary OBS to alleviate depressive symptoms in COPD patients, while highlighting areas for future research to strengthen evidence and applicability.

## 6. Conclusion

This study demonstrates that enhancing the OBS, particularly dietary OBS, could be an effective intervention strategy for mitigating depressive symptoms in patients with COPD. Optimizing dietary patterns (e.g., increasing antioxidant intake) and implementing appropriate lifestyle changes (e.g., smoking cessation and increased physical activity) may substantially improve both psychological and physical health. Future research should adopt longitudinal designs and experimental studies, incorporating oxidative stress and inflammation markers, to elucidate causal relationships and underlying mechanisms. Additionally, investigating the protective effects of OBS across comorbid conditions could provide valuable insights for integrated chronic disease management.^[40]^

## Acknowledgments

The author expresses gratitude to the NHANES study team members and participants for their indispensable contributions.

## Author contributions

**Data curation:** Ke Chen, Lu Zhang, Li Fang, Di Wu, Mengyao Shi, Zegeng Li.

**Formal analysis:** Ke Chen.

**Software:** Bowen Xu.

## Supplementary Material
