# Supplementary material for: Oxidative balance score and depression in COPD patients: A cross-sectional study from NHANES 2007 to 2018
Source: Medicine (Baltimore). 2025 Dec 12;104(50):e46004. doi: 10.1097/MD.0000000000046004 (PMC12708097; doi:10.1097/MD.0000000000046004)

Table S1. OBS Components and Scoring Criteria for Dietary and Lifestyle Factors

Component Category	Component	Property	Male	Female
Dietary OBS Components	Dietary Fiber (g/d)	Antioxidant	<12.60(0),12.60-21.10 (1), ≥21.10 (2)	<10.30 (0), 10.30-17.10 (1), ≥17.10 (2)
	Carotene (RE/d)	Antioxidant	<91.67 (0), 91.67-326.50 (1), ≥326.50 (2)	<88.33(0), 88.33-378.50 (1), ≥378.50 (2)
	Riboflavin (mg/d)	Antioxidant	<1.82 (0), 1.82-2.75 (1), ≥2.75 (2)	<1.36 (0), 1.36-2.09 (1), ≥2.09 (2)
	Niacin (mg/d)	Antioxidant	<21.91 (0), 21.91-33.06 (1), ≥33.06 (2)	<15.30 (0), 15.30-23.16 (1), ≥23.16 (2)
	Vitamin B6 (mg/d)	Antioxidant	<1.63 (0), 1.63-2.57 (1), ≥2.57 (2)	<1.17 (0), 1.17-1.88 (1), ≥1.88 (2)
	Total Folate (mcg/d)	Antioxidant	<319.00 (0), 319.00-506.00 (1), ≥506.00 (2)	<241.00 (0), 241.00-390.00 (1), ≥390.00 (2)
	Vitamin B12 (mcg/d)	Antioxidant	<3.43 (0), 3.43-6.39 (1), ≥6.39 (2)	<2.29 (0), 2.29-4.45 (1), ≥4.45 (2)
	Vitamin C (mg/d)	Antioxidant	<31.20 (0), 31.20-95.00 (1), ≥95.00 (2)	<29.70 (0), 29.70-85.50 (1), ≥85.50 (2)
	Vitamin E (ATE) (mg/d)	Antioxidant	<5.97 (0), 5.97-10.32 (1), ≥10.32 (2)	<4.84 (0), 4.84-8.22 (1), ≥8.22 (2)
	Calcium (mg/d)	Antioxidant	<697.00 (0), 697.00-1175.00 (1), ≥1175.00 (2)	<560.00 (0), 560.00-941.00 (1), ≥941.00 (2)
	Magnesium (mg/d)	Antioxidant	<257.00 (0), 257.00-376.36 (1), ≥376.36 (2)	<200.00 (0), 200.00-293.00 (1), ≥293.00 (2)
	Zinc (mg/d)	Antioxidant	<9.70 (0), 9.70-15.05 (1), ≥15.05 (2)	<6.94 (0), 6.94-10.79 (1), ≥10.79 (2)
	Copper (mg/d)	Antioxidant	<1.04 (0), 1.04-1.56 (1), ≥1.56 (2)	<0.84 (0), 0.84-1.25 (1), ≥1.25 (2)
	Selenium (mcg/d)	Antioxidant	<99.50 (0), 99.50-146.20 (1), ≥146.20 (2)	<70.00 (0), 70.00-106.20 (1), ≥106.20 (2)
	Total Fat (g/d)	Pro-oxidant	≥70.47 (0), 70.47-110.77 (1), <110.77 (2)	≥52.28 (0), 52.28-80.54 (1), <80.54 (2)
	Iron (mg/d)	Pro-oxidant	≥12.43 (0), 12.43-18.98 (1), <18.98 (2)	≥9.28 (0), 9.28-14.20 (1), <14.20 (2)
Lifestyle OBS Components	Physical Activity (MET-minutes/week)	Antioxidant	<1071.00 (0), 1071.00-3720.00 (1), ≥3720.00 (2)	<760.00 (0), 760.00-2480.00 (1), ≥2480.00 (2)
	Alcohol (g/d)	Pro-oxidant	≥30 (0), 0-30 (1), None (2)	≥15 (0), 0-15 (1), None (2)
	Body Mass Index (kg/m^2)	Pro-oxidant	≥25.70 (0), 25.70-29.98 (1), <29.98 (2)	≥24.84 (0), 24.84-30.80 (1), <30.80 (2)
	Cotinine (ng/mL)	Pro-oxidant	≥0.027 (0), 0.027-2.040 (1), <2.040 (2)	≥0.027 (0), 0.027-2.040 (1), <2.040 (2)

Notes:

1. **Dietary Components**: These components are classified as antioxidants or pro-oxidants. Antioxidants (e.g., vitamins C, E, selenium) are scored from low to high intake (0, 1, 2), while pro-oxidants (e.g., total fat, iron) are scored in reverse (0, 1, 2).
2. **Lifestyle Components**: Lifestyle factors (physical activity, BMI, alcohol intake, and smoking) are scored based on their role in oxidative stress. High physical activity, normal BMI, non-smoking, and low alcohol intake are associated with lower oxidative stress, while smoking, high BMI, and heavy alcohol intake are associated with higher oxidative stress.
3. **Scoring Method**: Most components are scored based on tertiles (divided into three equal groups by gender), with scores of 0, 1, or 2. Antioxidants are scored from low to high intake, while pro-oxidants are scored from high to low intake.

Table S2:Additional baseline characteristics based on depression risk in chronic obstructive pulmonary disease (COPD) patients

**Characteristic**	**Depression**		**p-value**
	**No** **Weighted N = 6,616,290** **Unweighted n = 851**[1]	**Yes** **Weighted N = 1,023,346** **Unweighted n = 169**[1]	
**Age**	58 (47, 65)	54 (46, 63)	0.120[2]
**Sex**			0.018[3]
Female	44.5%	57.3%	
Male	55.5%	42.7%	
**Race**			0.312[3]
Mexican American	1.5%	2.5%	
Non-Hispanic Black	6.7%	10.4%	
Non-Hispanic White	85.5%	78.9%	
Other Hispanic	2.1%	3.5%	
Other Race - Including Multi-Racial	4.2%	4.6%	
**Educational.attainment**			<0.001[3]
<High school	16.3%	33.3%	
College	58.8%	36.8%	
High school	24.9%	29.9%	
**Marital.status**			0.005[3]
Divorced/separated/widowed	24.2%	35.3%	
Married/living with a partner	66.6%	49.1%	
Never married	9.2%	15.7%	
**Poverty,n(%)**			<0.001[3]
< 1.3	20.0%	53.8%	
≥ 3.5	48.7%	16.3%	
1.3 - 3.5	31.3%	30.0%	
**BMI**	27 (23, 32)	28 (23, 34)	0.131[2]
**Total.energy.intake.kcal.**	2,047 (1,582, 2,726)	1,866 (1,220, 2,780)	0.102[2]
**HEI_2015**	20.00 (20.00, 21.50)	20.00 (20.00, 20.00)	0.008[2]
**CCI,n(%)**			0.547[3]
< 2	76.1%	73.0%	
≥ 2	23.9%	27.0%	
**OBS**	19 (13, 25)	15 (10, 19)	<0.001[2]
**Lifestyle OBS**	5.00 (4.00, 6.00)	4.00 (4.00, 5.00)	<0.001[2]
**Dietary OBS**	14 (9, 19)	10 (6, 15)	<0.001[2]

[1]Median (IQR); %

[2]Wilcoxon rank-sum test for complex survey samples

[3]chi-squared test with Rao & Scott's second-order correction

BMI: Body Mass Index,HEI: Healthy Eating Index,CCI: Charlson Comorbidity Index

TableS3 Results of multivariable logistic regression of OBS on depression risk after adjustment for extreme values.

Model	OBS coefficient (estimated value)	standard error (SE)	Z value	P value
Crude model	-0.08023	0.0148	-5.423	5.72×10^{-7}
Model 1	-0.05832	0.0156	-3.737	3.41×10^{-4}
Model 2	-0.08712	0.0182	-4.789	1.95×10^{-4}
Model 3	-0.08567	0.0179	-4.784	2.04×10^{-4}

Note：Model 1:Adjusted for age, sex, race, marital status, education level, and poverty-income ratio.

Model 2:Additionally adjusted for HEI, and total energy intake.

Model 3: Additionally, adjusted for CCI.

TableS4 Results of trend analysis of depression risk after OBS grouped by tertiles, quartiles and quintiles

Subgroup	Trend coefficient (estimated value)	standard error (SE)	Z value	P value
Quartile Analysis	-0.50505	0.09387	-5.38	9.08×10^{7}
Tertile Analysis	-0.40821	0.10128	-4.03	8.17×10^{6}
Quintile Analysis	0.52034	0.09503	-5.47	4.28×10^{7}

FigureS1.Forest Plot of OBS Coefficients Across Models

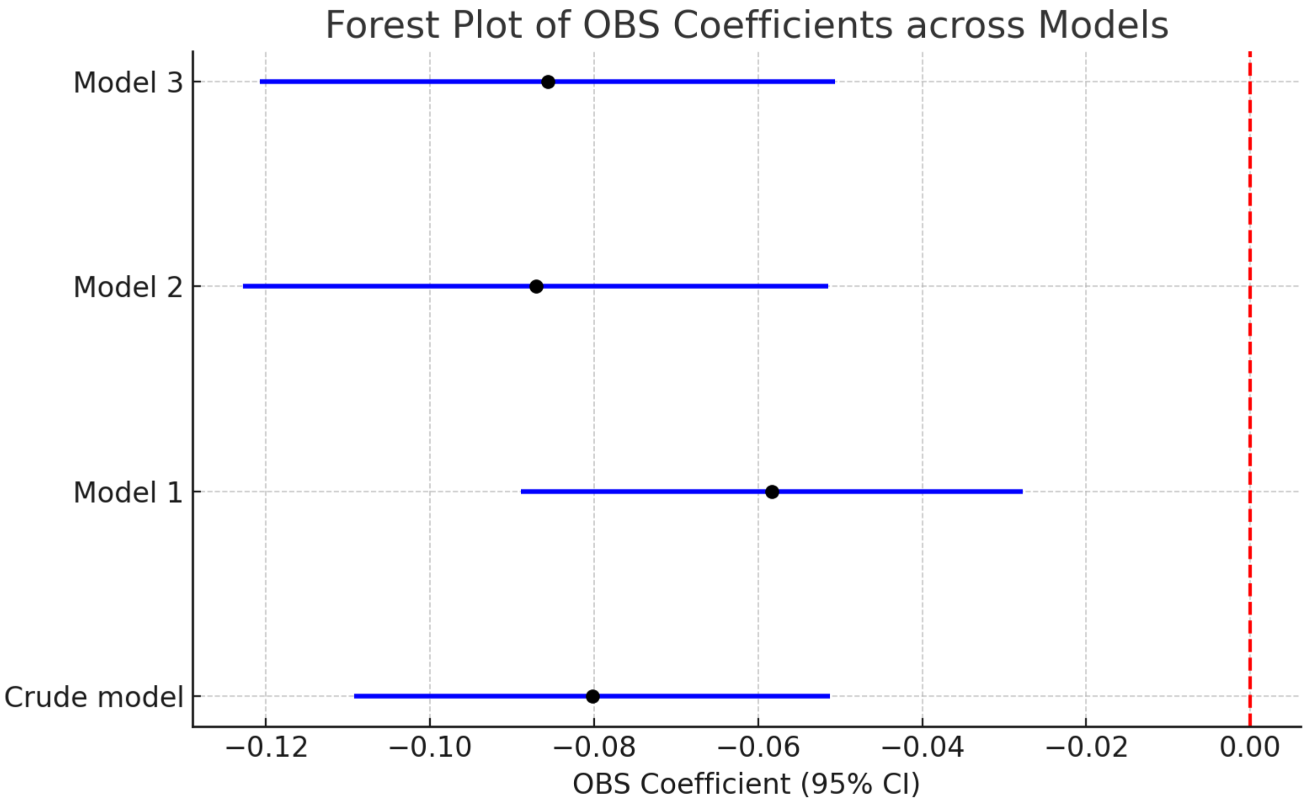

Supplement: Supplementary file 1 [file medi-104-e46004-s001.pdf]
